# Patients’ experience of using primary care services in the context of Indonesian universal health coverage reforms

**DOI:** 10.1186/s12930-017-0034-6

**Published:** 2017-03-21

**Authors:** Fitriana Murriya Ekawati, Mora Claramita, Krishna Hort, John Furler, Sharon Licqurish, Jane Gunn

**Affiliations:** 1 Department of Family and Community Medicine, Faculty of Medicine, Universitas Gadjah Mada, Yogyakarta, Indonesia; 2 Department of Medical Education, Faculty of Medicine, Universitas Gadjah Mada, Yogyakarta, Indonesia; 3 Department of General Practice and Primary Health Care, University of Melbourne, Melbourne, Australia; 4 Nossal Institute of Global Health, University of Melbourne, Melbourne, Australia

**Keywords:** General practice, Primary health care, Indonesia, Access, Patients’ experience, Phenomenology

## Abstract

**Background:**

The World Health Organization (WHO) recommendation on universal coverage has been implemented in Indonesia as *Jaminan Kesehatan Nasional* (JKN). It was designed to provide people with equitable and high-quality health care by strengthening primary care as the gate-keeper to hospitals. However, during its first year of implementation, recruitment of JKN members was slow, and the referral rates from primary to secondary care remained high. Little is known about how the public views the introduction of JKN or the factors that influence their decision to enroll in JKN.

**Aim:**

This research aimed to explore patients’ views on the implementation of JKN and factors that influence a person’s decision to enroll in the JKN scheme.

**Methods:**

This study was informed by interpretative phenomenological analysis (IPA) methodology to understand patients’ views. The interview participants were purposively recruited using maximum variation criteria. The data were gathered using in-depth interviews and was conducted in Yogyakarta from October to December 2014. The interviews were transcribed, translated and analyzed using IPA analysis.

**Result:**

Twenty three participants were interviewed from eight primary care clinics. Three superordinate themes: access, trust, and separation anxiety were identified which impacted on the uptake of JKN. Participants acknowledged that whilst primary care clinics were conveniently located, access was often complicated by long waiting times and short opening hours. Participants also expressed lower levels of trust with primary care doctors compared to hospital and specialist care. They also reported a sense of anxiety that the current JKN regulation might limit their ability to access the hospital service guaranteed in the past.

**Discussion:**

This study identified patients’ views that could challenge the implementation of the gate-keeper role of primary care in Indonesia. While the patients valued the availability of medical care close to home, their lack of trust in primary care doctors and fear that they might lost the hospital care in the future appears to have impacted on the uptake of JKN. Unless targeted efforts are made to address these views through sustained public education and further capacity building in primary care, it is unlikely that the full potential of the JKN scheme in primary care will be realized.

## Background

The WHO [1] has proposed that all member countries implement universal health coverage to help people access high-quality health services without financial barriers. This WHO recommendation of universal health coverage was in-line with the Alma Ata declaration which aimed to provide people with accessible high-quality services in primary care [2, 3]. Responding to those global recommendations, Indonesia has established a universal insurance scheme, which is known as *Jaminan Kesehatan Nasional* (JKN) since January 2014. The JKN was the merger of four pre-existing public insurance schemes (*Askes* insurance for civil servants, *Jamkesmas* insurance for poor citizens, *Jamsostek* as insurance for private sector workers and *Asabri* as Insurance for the armed forces). The JKN was designed under the law of National Social Security to meet the people’s basic needs and contribute towards the realization of a prosperous and equitable Indonesia [4, 5]. With this insurance, it was expected that people would not face financial barriers to access health care and therefore increase their use of health services.

The role of primary care as a gatekeeper to secondary and tertiary care has been strengthened in the JKN implementation. It is expected that primary care doctors can fulfill the people’s basic health needs and ought to be able to manage the majority of the patients’ problems [6]. In the JKN implementation, this sector is supported under a capitation payment system [7]. It is a payment concept within which the private family doctors and Puskesmas (Indonesian Public Primary Care Clinic) as primary care providers are paid a set payment per number of registered patients, whether the patients come to seek any medical help or not. This payment scheme is intended to enable primary care services to focus on health promotion and illness prevention in addition to curative measures [7].

The JKN also introduced several new regulations of the health system compared to the previous insurance schemes. Under the *Askes* and J*amkesmas* schemes in the past, patients were free to access any primary care service. They also could easily obtain a referral letter from their general practitioner (GP) to access secondary care in hospitals [8]. However, under the JKN scheme, patients have to formally register themselves at JKN offices or their appointed family doctor practice or Puskesmas. The guidelines of conditions that could be appropriately managed by the GPs-based on their competency standard [9] -had also been published [10]. The referrals could only be made on appropriate clinical judgements. Therefore, the patients have to comply with these procedures set by the JKN, under which, JKN will not cover the cost of any hospital treatment if the referral is made outside the guidelines [11, 12]. On the other hand, the Indonesian government also supported the utilization of primary care under the JKN by disseminating information on JKN procedures to the public and preparing practice guidelines for primary care providers. The information was spread by a massive media campaign including advertisements on TV and radio; while procedures were established to make registration with JKN easier for patients.

However, an additional policy that could contribute significantly to the success of JKN primary care objectives has yet to be fully established. This relates to the requirement for GPs to undertake compulsory formal training to improve their primary care practice. In 2015, 168,823 doctors were registered with the Indonesian Medical Council: 29,561 of them were specialist doctors, 29,665 were dentists, and 109,597 were recorded as GPs [13]. In Indonesia, doctors only need to undertake a four year undergraduate degree plus a two years postgraduate Medical Doctor (MD) degree, national examination, and one year internship in hospitals and Puskesmas to get the licence to practice as a GP in primary care [14]. During the undergraduate degree, the students learn the theory required to practise medicine. During the MD, they complete two years of clinical practice in various hospital departments; such as internal medicine, surgery, dermatology, and obstetrics. While the specialists have to complete an additional three to five years training at the University hospitals, currently, no compulsory training for GPs is available to upgrade their skills in primary care practice. Therefore, this level of training for Indonesian GPs differs from the requirements in other similar countries that are implementing universal insurance coverage. In Thailand for example, GPs have been required to undertake a formal postgraduate training in family medicine. This education scheme has been implemented since 20 years before the implementation of Thailand’s universal coverage in 2001 [15, 16].

Some important issues arose during JKN’s first years of implementation, notably slow recruitment of new members, which was identified at the first JKN mid-year evaluation. Compared to the total membership of the four previous insurances schemes in 2012 (155 million members), the JKN membership had only reached 125 million members in mid-2014 [10, 17]. The target of doctors to handle almost all of the primary care cases and only refer less than 10% of the cases was also not being met. Nationally, the overall JKN primary-to-secondary referral rate was 17%, but the referral rate in several provinces such as in Yogyakarta, East Java and Jakarta was much higher, up to 55% of cases [18, 19]. With this high referral rate, it is likely that many of the referrals were inappropriate, such as essential hypertension, dyspepsia, and general physical examination [11], conditions which should be managed at primary care level according to the Indonesian physicians’ competency standards for primary care doctors [9, 17].

Unfortunately, there has been little evidence available about the factors that underlie these emerging issues during the initial implementation of JKN in primary care settings. In particular, there is little known of patients’ views and experiences during JKN implementation, which may be important factors influencing their decision about whether to opt into the JKN. Information on the views of patients is important because patients are the actual users of health care, and their views and experiences represent the actual condition in practice settings [20, 21].

Current available evidence only shows that there are different satisfaction rates with the JKN program across Indonesian provinces. Gaghana, Siagian [22] found that only 51.9% out of 106 patients were satisfied with JKN implementation in Sulawesi. Putri [23] also found the implementation of JKN was not effective in primary care clinics in Padang city, West Sumatra. Putri found that the JKN improved service delivery for low socioeconomic status patients, but the patients expressed dissatisfaction with the staff responsiveness, credibility, medical documentation, and medical access. However, neither of these studies explored in depth the factors which contributed towards the patients’ views nor their dissatisfaction. Therefore, we set out to explore in depth the patients’ perspectives of primary care in this study, and the factors which contribute towards the slow recruitment and high referral rates to secondary care [10, 17].

## Methods

This research was informed by the interpretative phenomenological analysis (IPA) approach to allow a deep and comprehensive understanding of the participants’ views and experiences [24]. Consistent with this approach, data collection was conducted using semi-structured interviews to allow greater opportunity for patients to express their views.

The study was conducted in Yogyakarta province, central Indonesia, from October to December 2014. Yogyakarta is characterized by a range of socio-economic status of its population, relatively easy access to health services and a high rate of referral to secondary care. A maximum variation sampling strategy was applied to ensure a range of perspectives. The clinic recruitment process was done purposively in both private and public primary care clinics in the Yogyakarta region: at the districts of Kulonprogo, Sleman, Yogyakarta city, Bantul, and Gunung Kidul.

The recruitment process is described as follows: first, the practice manager/clinic owners were telephoned and were provided with *Bahasa Indonesia* plain language statement. When the practice manager/clinic owner had signed the consent form, first author (FM) then came to the clinics to recruit patients for an interview. The patients were selected purposively from the patients’ registry during the interview day (at maximum three participants at each clinic). The inclusion criteria included: Indonesian citizen, JKN insurance member, and Yogyakarta resident. Then, the patients were selected based on the following purposive sampling criteria: age, income level, the level of education, residential address, marital status, and specific characteristics; such as whether they were a parent or a caregiver. The only exclusion criterion was if patients were unwilling to participate in the research.

The first author (FM) completed all of the interviews. Written informed consent was obtained from all study participants prior to the interview. All of the interviews were done in a private room in the clinics, except two interviews which were done in FM’s office. None of the participants expressed any objection with the interview place. FM had also anticipated that Yogyakarta people may be reluctant to speak openly about their views [25] or may be hesitant because FM is a GP from Yogyakarta. FM therefore explained to the participants that their participation was voluntary, their answers were confidential, and that their participation would not affect their relationship with FM or with their care providers in the future.

During the interviews, FM began with an introduction. After that, she asked open-ended questions, such: *‘please tell me your experience in seeking health care’ or ‘where do you usually go to seek health care.’* Then, these questions were followed up with more focused interview questions related to the participant’s experience with the primary care service and the JKN implementation. The participant responses were probed and clarified using prompting sentences, summarizing sentences and or some positive statements such as *uhum…*, *or ‘yes ….*’ and silent pauses to allow time for participants to think about their responses and respond. All but one interview was tape-recorded. Written notes of responses were taken for the patient who was unwilling to be tape-recorded. All the participants were given a small souvenir bag as a token of their participation.

The interviews were transcribed and then, translated into English. Five translated texts were back translated for translation validation. The data were analyzed using IPA analysis. IPA is an analytical method to explore in depth the participants’ views, combined with the researcher’s interpretation of the participant’s meaning of a phenomena. The steps of IPA analysis were systematically applied according to the recommendations of by Smith and Osborn [26]. FM and JG read all the transcribed texts independently until they were also familiar with the patients’ views. They both then coded any notable quotes. The quotes were grouped into themes and super-ordinate themes. The emerging themes were discussed and crosschecked amongst other researchers with primary care backgrounds.

## Results

The proposed maximum variation sample design for interview participants was fulfilled. The criteria for the sample are listed in Table 1. All criteria were met with at minimum two participants in each category. 23 participants were recruited, the majority of whom were women (n = 17), had a high level of education (at minimum a bachelor degree) (n = 13), were aged between 26 and 65 years old, and of middle-income status/income per month: 1–5 million IDR (n = 12). The majority of participants were members of the previous insurance schemes, with 13 past members of Askes (civil servants scheme), and 6 were past members of Jamkesmas (free insurance for low-income/poor). Almost all the participants were willing to fully participate right up until completion of the interview. One participant suddenly decided to stop in the middle of the interview process but did not wish to withdraw completely from the study because of her worries that the interview would affect her treatment in the clinics.

**Table 1:** The participants’ characteristic

Sample criteria	Total number (N = 23)
Types of clinic	
Public clinic	5
Private clinic	3
Gender	
Male	7
Female	16
Age group	
18–25	4
26–45	8
46–65	7
66–85	4
Residential address	
Urban area	11
Rural area	12
Education level	
High education	13
Low education	10
Income level	
High income	6
Middle income	12
Low income	5
Employment sector	
Private sector	7
Public sector	16
Frequency use of primary care	
Never	4
1–5 visits/year	8
>5 visits/year	11
Marital status	
Married	18
Single	5
Diagnoses	
Acute illness	5
Chronic illness	9
Healthy	9
Specific attributes	
Only caregiver	–
Parent	13
Both of caregiver and parent	5
None	5

Three superordinate themes of Access, Trust, and Separation anxiety were identified from the analysis. Participants acknowledged the convenience of access to primary care but were dissatisfied with the waiting time and physical structure of the clinics. The superordinate theme of Trust referred to participants who were dissatisfied with the doctors’ general communication. They also expressed doubt that the primary care doctors could treat more severe diseases and preferred to receive a referral letter to secondary care. Within the third superordinate theme, participants expressed their anxiety about whether they would be able to continue to use specialist care services at the hospital in the way they were previously used to.

### Access

Many participants referred to the proximity between the service and their home and the convenience of access to primary care service and chose them as their usual means of health care. This answer also came from many participants when being asked about their reason for attending primary care. For instance, Participant 21 in this study said “*Yes, every month I go to Puskesmas. Because it is near, so that I can get the easily accessible service*” (Participant 21, l.24–25).

However, later on, during the interviews, many participants commented that the long waiting time and facilities in the clinics were less convenient and limited their enjoyment of the service. In some cases, the participants then preferred to leave the clinics or chose to attend a private hospital rather than continue queuing. For example, Participant 9 said: *“If in Puskesmas, I need to queue for a long time. I have to queue before here and there. But, I need to go to fieldwork (working), so I decide to leave the Puskesmas and go to private hospital” (Participant 9, l. 38*–*39).*

In addition, participants also noticed deficiencies in the physical facilities at the clinic which influenced the ease with which they could see the doctor. They considered that the clinic building could not accommodate them well and caused them to wait longer to see the doctors, as stated by Participant 11:

*“If all patients need and go to Puskesmas, I believe that it is not only unbalanced, but it is impossible. This Puskesmas does not have enough room for that, and we will wait longer and longer. The parking lot, Puskesmas also can’t provide an enough time for us (Participant 11, l.57*–*65).*

Some participants also commented on the facility inequalities across Indonesia’s geography. Participants 1 and 16 thought that service on the island of Java (where Yogyakarta is located) was actually better than the service in other islands. They felt that although JKN had already helped people with affordable medical cost, it still did not resolve the inequalities and hindered access to high-quality medical care for people residing outside Java, due to the imbalanced and non-standardized health facilities in different geographic areas.

*“Who gets the benefits, I think, once again are Javanese, Can you imagine? In East Nusa*-*Tenggara, on the top of the mountain, a member of JKN, she needs a bypass of her heart, can she? Because they are people who cannot be served, they should have the same rights; they should get the same, but in fact, they receive different service. Big fake as long as the government does not think about the infrastructures, doctors’ distribution, hospitals and the nurses.” (Participant 1, l.182*–*186).*

### Trust

Participants were initially unsure when asked about the quality of the primary care doctors’ service. They usually answered with comments such as *‘the doctors are kind’* or ‘*I feel comfortable with the doctors*’ as their first reply. However, when they were encouraged to give more description about what they expected from their GPs, they expected that GPs should have excellent communication skills and be able to explain more about the patients’ condition. For instance, Participants 11 and 12 were dissatisfied with the doctors’ service because the doctors did not explain more about their illnesses. They thought that the doctors only gave similar pills for all patients without adequate information. They expected the GPs to perform a comprehensive individual examination and provide a complete explanation of their condition so that they understood the purposes of the medication and trusted the doctors’ treatment.

*“So usually in Puskesmas, you see that after the doctor asks us, then he will give us some medicine? Then we do not know what is our disease is. So I think the doctor only treat our symptoms. So my hope is that we are asked comprehensively. So that we know our problem, not only fever, then the doctor gives us the fever drug. However, then we develop an infection and not yet informed. Everyone is given antibiotic*”. (Participant 12, l. 68–72).

Another prominent aspect of patients’ trust in their GPs is that many participants also doubted if the GPs could manage more severe problems. They considered that the GPs’ current education could only equip them with skills to superficially understand the patients’ conditions. Many of them expressed the opinion that their GPs were unable to solve more serious illnesses, as was explained by Participant 8:

*“The GP is a general doctor, their education is limited. I think for the specific diagnosis, the internist’s (diagnosis) is better, specific in the treatment as well. The GP doctors, I believe they need to learn again”* (Participant 8, l. 61–62).

Furthermore, some participants insisted that the GPs’ role should be to provide referrals to hospital. There was a perception among patients that if a person was suffering from any illness, they needed to see a specialist and the primary care doctors should give them referrals to hospital, as expressed by Participant 16:

*“I usually get referrals from here. A long time ago, I must be inpatient in S hospital; I got the referral from here. I also once had a referral from here for a urinary infection. When my child was sick, I came here. Sometimes I think the primary care is not needed, but the pediatrician. My child’s eyes were red and got swelling several times, which we are referred to J hospital. My wife also comes here, my mother in law also gets the referral here” (Participant 16, l. 41–48)”.*

Meanwhile, participants also considered that specialist doctors were also superior because of their more comprehensive facilities. Participant 6 stated that she was pessimistic with the clinic service because it had less medical facilities. She said that this made the GPs use less effective medication and limited the help they could provide for the patients’ problems, whereas, specialist care was always trusted as it was always complemented with more advanced medication. Interestingly, Participant 6 also believed that diseases of certain body parts belonged to certain specialist expertise. She thought that as nowadays many patients suffered from internal diseases. She then expected the clinic to have an internist doctor.

*“I think it will be good if you can provide an ophthalmologist because you know, it is a pity for many people here with cataract, an eye*-*wart, an eye spot, sometimes ago, they were here and could not be treated. For eye diseases in Puskesmas, we were only given an eye drop. I do not think Puskesmas can give anything else for cataract patients, for now, I do not know for the future. But with the eye drops, when I used it, I do not feel any improvement, Somewhat my eyes are getting bright but often darker, it is not working. Moreover, please, you may add this clinic with an internist. You know there are lots of patients with internal diseases”* (Participant 6, l. 78–90).

### Separation anxiety

Thus, with the JKN transition, half of the participants in this study experienced JKN implementation in primary care as a challenging period because they no longer had the same access to the specialist services, which they regarded as superior to primary care-that they had been guaranteed in the past. Many participants, who had been managed by specialists for a long time, now had to visit the GPs for their routine care. They also claimed that JKN restricted the prescription of medicines and their referral access to the hospital. As they already had limited trust in the GP’s ability (-as described at ‘Trust’ theme), patients worried if the GP care was less ideal than specialist care as expressed by Participant 2:

*“Well, this is just my opinion. You know that I should be referred to an internist because I have diabetes. Previously, I was happy in G clinic (specialist clinic), I could see the internist directly. You know if here, I have to use the referral system, which is once in every three months in the hospital. I need to see the GPs for months. I think that is too long. However, anyhow, I kept ask my doctor to refer me to specialist and now the doctors refer me”* (Patient 2, l. 49–52).

Participants were more emotionally discouraged because JKN had also limited some hospital services which had previously been covered by *Askes* insurance. Even though they had already had referrals to the hospital, it did not mean that JKN would cover the all the hospital medical expenses. Participant 21 mentioned that some services were not fully covered by JKN anymore, compared to *Askes* insurance in the past. She also expressed her feeling that somehow she was happier with the old *Askes* insurance.

*“I think with Askes if we come to health facilities whatever whenever, were not limited, but now, we have our limit. However, last month when my son had surgery, he should pay himself. It is stated at JKN paper that it is only covered for 15 million so that he had to pay the gaps. You know, it would not happen in Askes. That was different between them. For JKN, if the patient is not very ill and she is referred hospital, it will not be covered. We have to pay, come to the emergency room and pay. However, Askes was not. For me, they are different. I am happier with Askes because we do not have our limit”. (Participant 21, l. 72*–*79).*

Moreover, half of the participants also complained that clear information regarding JKN procedures was limitedly available. They were unfamiliar with current JKN transition systems in primary care. Even though there was increasing efforts to promote the changes that had been made, access to information in the primary care setting was lacking. Moreover, the clinic’s staff failed to assist patients in obtaining adequate information. For example, Participant 1 told of his experience in using a referral to access hospital care without knowing that the regulations were changing. He was dissatisfied because his referral to the hospital was no longer allowed and this was not informed by the clinic’s staff. He was concerned that the same situation could be experienced by another patient who needed urgent medical help.

*“I came at noon, Oh My God, the hospital officer showed me that referral was not working. She told me that the regulation was changing, I should go to the secondary hospital first. I mean, how about the other patients, if he was certain with the referral, but it did not work? How about if there is a severe patient, more serious than me, how is that?”* (Participant 1, l. 107–112).

## Discussion

Our findings suggest that during the commencement of the JKN universal health coverage scheme, Indonesian primary care faces some challenges in achieving the aims of the program. The limitations in facilities and operation have constrained the fulfillment of the patients’ perceived general health needs and reduced the public’s expectations regarding the JKN implementation in primary care.

The findings regarding the clinics access are consistent with existing research. Putri [23] found that patients reported inadequate access to primary care clinics during JKN insurance. Regarding this finding, one strategy to improve the facilities and access standard of care by providing an equal access of health care distribution [27] and establishing an accreditation program so that the care quality for the patients is warranted [28].

Even though still a distance from the ideal views of those suggestion, however, the health care distribution is currently the Indonesian priority. The government has currently established an accreditation standards required for primary care practice. The Puskesmas and private clinics that wish to contract with the JKN are expected to prepare and maintain their facilities to the required, specified standards of care, such as the fixed opening hours and patient care standard operating procedures. However, this program is currently ongoing and focusing on the Puskesmas [29], not yet involving the private primary care practices accreditation. The health care providers availability is also continuing to improve through several programs, such as contract doctor, Nusantara Sehat [30] and local public agency program which enable Puskesmas to arrange and manage its own facilities [31].

Another important finding in this research is that patient perceptions of the quality of the GPs practice also significantly influenced their trust in primary care settings. While the JKN seeks to establish GPs as gate—keepers in primary care, patients expressed different views on the GP practice. Some key elements of primary care, such as adequate communication, were less prominent in comparison to the patients’ comments that the GPs should refer them to secondary care [32]. This finding about the GPs care complemented another study findings on GPs experience that the they were currently focused on restricted policies in primary care practices and limitedly covered the trust issue between them and the patients [33].

Our result showed that the patients’ limited trust in GPs was very likely correlated with their views on the current doctors’ education that only enabled them to manage mild illnesses as what had been said by Participant 8. This finding is new and limitedly discussed in Indonesian literature. Meanwhile, the patients’ trust to their GPs was an important factor that contributed towards the high referrals from primary care [17]. Likewise, research in Central Asia [33], China [34] and Thailand [35–38] showed that the GPs’ roles were diminished because patients put less trust in them than specialists because of their shorter training. One strategy that could help build the patients’ trust in GPs in these countries is to include family medicine education as a compulsory element of GPs’ postgraduate training. The training could focus on preparing GPs to perform person-centred communication and to manage common cases in primary care [33, 39]. In Thailand, this training was successful in equipping the GPs to improve their gate-keeper role during the implementation of Thailand’s universal coverage scheme. The rate of inappropriate referral to secondary care could be minimized, patients could benefit from more appropriate care and the health financing system could be more efficient [16, 38].

Fortunately, the Indonesian government has also currently proposed such family medicine training for the GPs, but the implementation is still pending. Primary care training for GPs is already included in the Indonesian Medical Education Act number 20 the year 2013 [40]. The Indonesian National Board of Primary Care Physicians’ which brings together representatives of 17 major faculties of medicines has been preparing the formal postgraduate training for the GPs. However, there has been an extensive debates among GPs’ and specialist colleagues regarding the training time, resources, and care collaboration. Concerns have been raised that the postgraduate training would lengthen the GPs’ education to practice in primary care and that the training would be ineffective without ensuring adequate facilities were available in the primary care settings [41]. Therefore, recognition of prior learning (RPL) has been offered as an alternative, so that the current GPs will still be able to practice while they have the training. Unfortunately, until now, the debate is continuing and is prolonging the delay to establish a formal GP training. This delay could hinder improvement in the quality of practice and prolong the uncertainty among patients on the quality of primary care services [42].

Besides by upgrading the GPs capability, there is also a need to establish a clinical pathway guidelines [27]. This guideline would be very important as the GPs reference to provide a comprehensive care for their patients and manage the patients’ referrals. Even though the Indonesian Ministry of Health has published the Primary Care Guideline for GPs in Primary Care [43], its improvement is needed, particularly to provide the a care collaboration for re-referral mechanism between GPs and specialist in secondary care.

In addition to the factors related to the primary care access and the GPs care discussed above, the patients’ experience during JKN implementation in this study was also influenced by their experience with previous insurance schemes. Patients were unaware of the JKN changes and frequently compared their JKN experience with *Askes* (former scheme for civil servants) insurance, so that when the JKN strictly regulated some aspects of care, patients experienced dissatisfaction. Unfortunately, the available information failed to assist the patients in understanding the aims of JKN transition and the new role of primary care service. Therefore, a more extensive public information campaign in television, radio, or at any community meetings is essential so that people can understand what is JKN, what is covered, what is not covered, and what are the GPs’ roles in the JKN scheme. While the literature showed that GPs also felt overwhelmed with the JKN working load [44], an insurance specialist may also help to inform the patients about any JKN regulation changes at the clinics settings.

## Conclusion

In conclusion, this study filled in the gaps of literature of patients’ views about the implementation of JKN in primary care. This study concludes that the objectives of universal coverage in primary care have not yet been fully realised in Yogyakarta. To strengthen the JKN implementation, a change in public attitudes about universal coverage and the role of primary care practice is required. The public’s preference for hospital care, and trust in primary care, could be shifted by a better understanding of the benefits of primary care services. Those changes, obviously need the collaboration of all the parties involved in the JKN transition, particularly to support the primary care sectors to provide a high-quality service for patients, including the role of media to support the dissemination of information. Unless these issues of primary care are addressed, universal care will be difficult to achieve, and the public medical expense will remain high with inappropriate expense paid for unnecessary procedures in secondary and tertiary care [6, 16, 45].

This research has also provides a foundation for further deep investigation into doctors’ views and experience practicing with the Indonesian JKN as well as the Indonesian people’s opinions about postgraduate training in primary care/family medicine.

## Strengths and limitations

This study was a qualitative study with a relatively small sample of participants from Yogyakarta, and the findings should be interpreted and applied within the appropriate context, and might not represent the full range of donesian geographic diversity. The recruitment process was able to achieve data collection from a range of sources, and we are confident that our strategy to analyze the data using interpretative phenomenology has strengthened the findings and conclusions.

## Appendix

See Table 2.

**Table 2:** Detailed background of participants

Participants order	Clinics origin	Clinic characteristic	Gender	Age range	Education level	Income level	Residential address	Previous insurance coverage
1	Clinic 1	Private	Male	26–45	High	Middle	Urban	None
2	Clinic 2	Public	Male	46–65	Low	Middle	Rural	Askes
3	Clinic 1	Private	Male	18–25	High	Middle	Urban	Askes
4	Clinic 3	Private	Female	18–25	High	Middle	Rural	None
5	Clinic 4	Public	Male	46–65	Low	Middle	Rural	None
6	Clinic 4	Public	Female	46–65	Low	Low	Rural	Jamkesmas
7	Clinic 4	Public	Female	46–65	Low	Low	Rural	Jamkesmas
8	Clinic 3	Private	Male	46–65	High	High	Rural	Askes
9	Clinic 3	Private	Female	26–45	High	Middle	Rural	Askes
10	Clinic 5	Public	Female	18–25	High	Middle	Urban	Askes
11	Clinic 5	Public	Male	26–45	High	High	Urban	None
12	Clinic 5	Public	Female	18–25	High	Middle	Urban	Askes
13	Clinic 6	Public	Female	26–45	Low	Low	Rural	Jamkesmas
14	Clinic 6	Public	Female	26–45	High	Middle	Rural	Askes
15	Clinic 2	Public	Female	26–45	High	High	Urban	Askes
16	Clinic 2	Public	Male	26–45	High	High	Urban	Askes
17	Clinic 1	Private	Female	66–85	High	High	Urban	Askes
18	Clinic 7	Public	Female	66–86	Low	Low	Rural	Jamkesmas
19	Clinic 7	Public	Female	46–65	Low	Low	Rural	Jamkesmas
20	Clinic 7	Public	Female	26–45	Low	Middle	Rural	Jamkesmas
21	Clinic 8	Private	Female	66–86	Low	Middle	Urban	Askes
22	Clinic 8	Private	Female	66–87	Low	Middle	Urban	Askes
23	Clinic 8	Private	Female	46–65	High	High	Urban	Askes

High education: minimum undergraduate degree, low education: maximum senior high school; high income: average income per month above 5.000.000 IDR, middle income: Average income per month 1.000.000 IDR–5.000.000 IDR, Low income: average income per month less than 1.000.000 IDR; Rural area: living in a rural area (Respondents live more than 20 km from the CBD), Urban area: living in a urban area (Respondents live within a 20 km radius from CBD)

## Abbreviations

Askes: Asuransi Kesehatan (Insurance for Indonesian civil servants)
GP: General Practitioner
Jamkesmas: Jaminan Kesehatan Masyarakat (Insurance for the poor citizens)
Jamsostek: Jaminan Sosial Tenaga Kerja (Insurance for workers)
JKN: Jaminan Kesehatan Nasional (Indonesin universal health coverage)
IPA: Interpretative Phenomenological Analysis
Puskesmas: Pusat Kesehatan Masyarakat (Indonesian public primary care clinics)
